# Applying machine learning models to predict and identify factors affecting academic performance of paramedical students: a cross-sectional study

**DOI:** 10.1186/s12909-026-08799-3

**Published:** 2026-02-19

**Authors:** Omid Zarei, Maryam Talebi Moghaddam, Zahra Arefzadeh, Sadegh Moradi Vastegani, Fatemeh Zeraatpishe, Najimeh Beygi, Mohammad Ghorbani

**Affiliations:** 1 Department of Anesthesiology, School of Allied Medical Sciences, Fasa University of Medical Sciences, Fasa, Iran; 2 Noncommunicable Diseases Research Center, Fasa University of Medical Sciences, Fasa, Iran; 3 Department of Computer Science, Engineering and Information Technology, School of Electrical and Computer Engineering, Shiraz University, Shiraz, Iran; 4 Medical Plants Research Center, Basic Health Sciences Institute, Shahrekord University of Medical Sciences, Shahrekord, Iran; 5 Persian Gulf Physiology Research Center, Medical Basic Sciences Research Institute, Ahvaz Jundishapur University of Medical Sciences, Ahvaz, Iran; 6 School of Allied Medical Sciences, Fasa University of Medical Sciences, Fasa, Iran; 7 Department of Critical Care Nursing, Fasa University of Medical Sciences, Fasa, Iran; 8 Department of Pathology, School of Allied Medical Sciences, Fasa University of Medical Sciences, Fasa, Iran

**Keywords:** Academic performance, Cross-Sectional study, Feature importance analysis, Higher education, Machine learning, Random forests

## Abstract

**Background:**

Academic performance is a key indicator of student success and institutional effectiveness in higher education, especially in paramedical fields where precision and competence are essential. Yet traditional methods often miss these complex factors. This study utilizes machine learning to predict performance and identify key influences among paramedical students, providing data-driven insights to inform the improvement of educational strategies.

**Methods:**

We conducted a cross-sectional study among 135 paramedical students at Fasa University of Medical Sciences, Iran, using convenience sampling. The dataset was constructed by combining face-to-face, paper-based self-administered questionnaire responses with students’ academic records obtained from the university’s Central Education Office. Validated questionnaires assessed demographics and failure/success factors. We applied Random Forest, Decision Tree, and Gradient Boosting models to predict outcomes and estimate feature importance. Model performance was evaluated using accuracy, precision, recall, F1-score, AUC, and G-mean with an 80:20 train–test split, and results were averaged over 10 iterations.

**Results:**

The Random Forest model excelled at predicting academic failure, achieving an accuracy of 90.74%, an F1-score of 76.19%, and an AUC of 96.19%, highlighting its precision in identifying at-risk students. Conversely, Gradient Boosting outperformed in predicting academic success, with an accuracy of 90.74%, an F1-score of 94.25%, and an AUC of 93.45%, demonstrating its ability to recognize improvement trends effectively. High school GPA emerged as the most important predictor of both outcomes, followed by academic traits and educational factors. Exploratory decision tree visualizations indicated possible hierarchical interactions, such as those potentially linking regional quotas and field of study to failure risk, and gender to success pathways; however, given the instability of decision trees on small datasets, these patterns are preliminary and require validation in larger cohorts. This study advances the application of machine learning in educational research, providing actionable insights for targeted interventions and policy refinement in paramedical education.

## Introduction

Academic failure is a significant challenge for universities, as it not only leads to the inefficient use of time and resources but also contributes to broader issues, including psychological, familial, and social difficulties. These problems escalate every year to the extent that many students struggle to cope with their studies and ultimately drop out of the university [1]. Research indicates that approximately 50% of incoming university students encounter academic underperformance during their first year [2]. Academic failure encompasses various aspects of educational failure, including frequent absences from classes, dropping out, repeating a grade, or receiving a low-quality education [3]. UNESCO (United Nations Educational, Scientific, and Cultural Organization) attributes the concept of academic failure to grade retention, early withdrawal from education, and a failure in the educational quality of learners [4]. Although definitions of academic failure vary, they uniformly highlight an inability or failure to complete formal education [5]. In the present study, to ensure clarity and consistency, academic failure and academic success/progress were operationally defined using official academic records from the University’s Central Education Office across two consecutive semesters. Academic failure was defined as a decrease of at least two GPA points compared with the previous semester together with a GPA < 14, whereas academic success/progress was defined as a GPA ≥ 15 accompanied by an increase in GPA compared with the previous semester. These categories were mutually exclusive; borderline cases (14 ≤ GPA < 15) and cases with unchanged GPA across the two semesters were not included in the labeled analyses. However, one of the most serious issues is the high rate of student failure in higher education, especially during the first year of study [6]. The results of studies provide evidence that various determinants influence students’ academic performance throughout the educational process [6–8]. In this regard, factors such as morale, intelligence, behavior, motivation, friends, job, parents’ education level, economic and social status, GPA, and student admission quotas [9, 10], psychological and personality issues, social and demographic tendencies, cognitive problems, self-efficacy, and the educational environment have been influential in students’ academic performance [11]. On the other hand, Dante and colleagues conducted a systematic review to evaluate the factors affecting student success and failure. The research findings indicated that gender, age, group affiliation, students’ personality traits, students’ perceptions of their major, family commitments, learning environment, and students’ performance were variables affecting student success and failure [9].

The opposite of academic failure is academic success and progress, which York and colleagues define as student advancement, satisfaction, skill and competency acquisition, perseverance, achievement of learning goals, and career success [12]. Academic progress is one of the primary factors that employers consider when recruiting the workforce, especially recent graduates. Therefore, students must put forth their utmost effort to achieve good grades to meet employers’ expectations [13]. Since students are the primary assets of universities and institutions, their performance during their studies plays a significant role in producing quality graduates [13–15]. Therefore, predicting student performance is crucial for providing them with the necessary assistance in the learning process [16]. Universities have initiated continuous and numerous evaluation processes to identify fundamental problems in the area of student academic performance and the quality of educational services provided [6]. Regarding the investigation of the causes of student academic failure and progress, most existing research has been limited to analyzing and predicting student performance by formulating problems straightforwardly and using limited statistical techniques [17].

There is a perceived need to conduct more precise investigations of student academic performance by employing modern data analysis methods [18]. Recent survey evidence highlights the rapid growth of educational data mining and machine learning methods for predicting students’ academic performance and supporting early interventions [19].

In this regard, machine learning techniques can predict student performance based on specified classified features and can be utilized by both students and academic institutions [20].

Consistent with this, machine learning has been used for early detection of students at risk of academic failure, enabling proactive support [21]. Consequently, this study was conducted to use machine learning-based models to predict the factors influencing success and academic failure among students at the Faculty of Paramedicine at Fasa University of Medical Sciences in southern Iran. Various machine learning classification methods were evaluated to identify the most effective algorithms for predicting students’ academic progress or failure.

### Related work

Although numerous studies have investigated the use of machine learning algorithms and educational data mining to predict factors affecting students’ academic improvement and failure, to the best of our knowledge, the innovation applied in this study has not been reported in prior research. This is considered a key strength of the present study. Nevertheless, in this section we review the most recent and relevant studies related to our work. Data mining techniques and machine learning algorithms can be applied in educational fields to enhance our understanding of learning processes by focusing on identifying, extracting, and evaluating variables that influence students’ learning [22, 23]. Kolo et al. (2015) conducted a study to apply a decision tree to analyze the academic performance of students in Nigeria. The study revealed that students’ financial status, motivation to learn, and gender were among the most significant factors influencing their academic performance. The study also predicted that 66.8% of students would complete their course, while 33.2% would not be able to finish. The researchers emphasized that prioritizing the use of more predictive models and analyses is essential in educational systems [24].

Lau et al. (2019) conducted a study aimed at modeling, predicting, and classifying students’ academic performance in China using artificial neural networks. The data collected for this research were based on socioeconomic factors and the results of the university entrance exams for undergraduate students. The results of this study demonstrated that the neural network model was able to predict academic performance with an accuracy of 84.8%. The researchers also emphasized that future education, through AI-based analysis, can enhance students’ ability to achieve high academic performance [17]. Mengash (2020) conducted a study in Saudi Arabia to apply data mining techniques for predicting student performance, thereby supporting decision-making in university admission systems. Four data mining models were employed in the survey: Artificial Neural Network, Decision Tree, Support Vector Machine, and Naive Bayes.

The results revealed that the Artificial Neural Network (ANN) model had the highest prediction accuracy at 79.22%, followed by the Support Vector Machine, Decision Tree, and Naive Bayes models, with accuracies of 75.91%, 75.82%, and 73.61%, respectively. This research contributed to the institution by helping to modify the admission criteria based on the proposed model, with a focus on selecting high-performing students in the early stages. Ultimately, the study reported that the new admission criteria resulted in a 31% improvement in.

overall performance and an 18% reduction in the percentage of underperforming students [25]. Sandoval et al. (2020) conducted a study in Spain aimed at training an artificial neural network model to predict student failure during their academic course. The variables examined in the study included demographic, academic, and socio-economic factors.

Using 12 student-related variables, an artificial neural network model was trained. The results indicated that this model could serve as a guide for shaping intervention policies to determine the likelihood of student failure during academic-level courses [6]. Butuner et al. (2022) conducted a study at Ankara University in Turkey aimed at predicting student academic performance in distance education programs using data mining methods. The study utilized a variety of techniques, including Naive Bayes, Random Forest, Support Vector Machine, K-Nearest Neighbors, Logistic Regression, Artificial Neural Network, and Deep Learning, to analyze data from learning management systems related to distance education programs. They used a predictive model that classified the results of data analysis into three levels: low, medium, and high. The researchers found that among all the methods mentioned, Deep Learning, Random Forest, and Support Vector Machine algorithms performed the best in predicting learner success [26].

In addition to these regional and model-comparison studies, recent internationally recognized work has emphasized (i) evidence synthesis across broader EDM research, (ii) early-warning systems that combine strong predictive performance with explainability, and (iii) bias-aware evaluation across student subgroups. Batool et al. (2023) provide a survey of student performance prediction and compare approximately 260 studies over the last 20 years in terms of major predictive factors, prediction and feature selection algorithms, and commonly used tools. Their analysis reports that ANN and Random Forest are among the most frequently used algorithms, WEKA is identified as a trending tool, and students’ academic records and demographic factors are among the strongest attributes for predicting performance. They also report that irrelevant features reduce prediction results and increase model processing time, and therefore note that almost half of the reviewed studies used feature selection techniques before building prediction models [19]. In the context of early detection of failure, López-García et al. (2023) propose an intelligent system to predict academic failure using student information stored by the Industrial University of Santander (Colombia). Their prediction model is powered by XGBoost and includes a TOPSIS-based feature extraction stage and ADASYN oversampling, with hyperparameters tuned by a cross-validated grid-search algorithm. They compare results with other decision-tree classifiers and report an explainability phase by displaying feature importance; in their conclusions, economic, health, and social factors are presented as decisive for students’ academic performance in their setting [21]. Finally, recent research has highlighted that prediction quality may vary across student groups, raising important concerns for responsible deployment. Herrmann and Weigert (2024) explore the extent to which predictive accuracy of academic success varies between traditional and non-traditional students (NTS). In their case study, they compare several popular algorithms and report that the accuracy of predicting academic success for NTS is significantly lower than when considering all students as a whole; they also note that the direction of distortion cannot be determined exactly due to small case numbers. The study emphasizes that potential bias must always be considered when predicting study success and that the use of such tools must ensure there are no undesirable biases that could affect certain students [27].

These studies suggest that data mining techniques and machine learning algorithms play a crucial role in analyzing and enhancing students’ academic performance and in identifying at-risk learners. Recent internationally recognized works further highlight the importance of systematic evidence synthesis, explainability in early-warning systems, and bias-aware evaluation across student subgroups. The present study, with its special focus and innovations, makes a valuable contribution to this scientific field.

### Design and study population

The present study is a cross-sectional study in which participants were selected using a convenience sampling method from among the students of the Faculty of Paramedical Sciences at Fasa University of Medical Sciences in southern Iran. A total of 135 students from the fields of anesthesia, operating room technology, and laboratory sciences participated in this study during the first semester of 2024. These students were in their second, third, and fourth years of study. 55 (40.74%) of the participants were men, and 80 (59.25%) were women.

At the outset of the project, a member of the research team collaborated with the University’s central Education Department to meet with students from the fields of anesthesia, operating room technology, and laboratory sciences. During this meeting, the researcher explained the study’s objectives and provided detailed instructions on completing the questionnaires to students interested in participating. This ensured that all participants understood the purpose and methodology of the research. One week after the questionnaires were distributed, the researcher returned to the educational groups (anesthesia, operating room technology, and laboratory science) to collect the completed questionnaires. This timely collection ensured that all data were collected efficiently and effectively, facilitating the next steps in the research process.

It is worth noting that the academic success questionnaires were administered to students who demonstrated academic success, while the academic failure questionnaires were administered to students who experienced academic failure. This was done in full compliance with ethical principles to ensure that during the distribution of the questionnaires, participants with academic failure could not be identified by others.

Importantly, the two questionnaires were parallel forms measuring the same constructs with identical domain structure; the ‘failure’ form used negatively worded equivalents of the same items to reduce stigmatization during administration. Prior to analysis, negatively worded items were reverse-scored, and domain scores were computed identically across all participants to ensure comparability. Using academic records obtained from the University’s Central Education Office, participants were categorized into two groups based on their academic status across the previous two semesters. These operational definitions were based on the official educational bylaws applied in Iranian universities affiliated with the Ministry of Health and Medical Education (MOHME) and were implemented in practice by the University’s Central Education Office to identify academic decline and academic progress across consecutive semesters. The first group comprised students with academic failure (a decrease of two points compared with the previous semester and a GPA < 14), and the second group comprised students with academic success/progress (a GPA ≥ 15 and an increase in GPA compared with the previous semester). These definitions are mutually exclusive, and a student cannot simultaneously meet the criteria for academic failure and academic success. To avoid ambiguity in labeling, borderline cases with 14 ≤ GPA < 15, as well as cases with unchanged GPA across the two semesters, were excluded from the labeled analyses. Accordingly, the final labeled dataset included 103 “success” instances and 32 “failure” instances (total *n* = 135).

In the continuation of the study, the collected data (from the questionnaire and the University’s Central Education Office) were analyzed using machine learning algorithms to predict the factors affecting students’ academic failure and success. The most effective factors were then extracted. Finally, it was provided to the Faculty of Paramedical Sciences to enhance the quality of student education, empower officials to make managerial and educational decisions, and implement practical changes.

### Data collection

Part of the data for this study was collected through three questionnaires (demographic information, academic failure questionnaire, and academic success questionnaire). The first questionnaire was related to the demographic information of the students, which included the variables of sex, age, marital status, field of study, year of university entry, time interval between receiving a diploma (gap year) and entering university, residence status, diploma grade, total academic grade in university, employment status, and university entry quota. The second questionnaire was the Academic Failure Questionnaire, which examined the factors affecting academic failure from the student’s perspective. This questionnaire is based on a 5-level Likert scale, consists of 59 items, and has three sections that measure individual, educational, and social factors affecting academic failure, respectively.

The third questionnaire was the Academic Success Questionnaire, which examined the factors affecting academic success from the student’s perspective. This questionnaire was also designed using a 5-level Likert scale and included 59 items, measuring individual, educational, and social factors that affect academic success.

To minimize stigmatization, we administered two parallel questionnaire forms (success vs. failure) with conceptually matched items (e.g., ‘having motivation’ vs. ‘lack of motivation’, ‘parents’ attention’ vs. ‘parents’ lack of attention’). Negatively worded items were reverse-coded prior to computing the aggregated domain scores (personal/academic/social/educational), so that higher scores consistently reflected higher levels of each construct across both forms.

The validity and reliability of these two questionnaires have been examined by Davarinia et al. in Iran [28]. Faculty members confirmed its validity, and the reliability of the entire instrument was measured and evaluated using the Cronbach’s alpha method. The Cronbach’s alpha coefficient for these two instruments was reported to be higher than 0.7, which is a desirable value. Another aspect of the data in the present study pertained to the academic status of students in the Faculty of Paramedical Sciences. This data was obtained from the Central Education Department of Fasa University of Medical Sciences, with permission from the university’s ethics committee and written consent from the students participating in the research. This data included the average score of all students during the past two semesters. It was used to identify students who were at risk of academic failure and those who were successful.

### Inclusion and exclusion criteria

Participants were eligible if they were students enrolled in paramedical fields and had completed at least two semesters of university coursework (i.e., second-year students or higher). Students were excluded if they were guest or transfer students, submitted an incomplete questionnaire, or expressed unwillingness to continue participation at any stage of the study.

## Method

In this section, we describe the machine learning-based approach used to predict and analyze factors influencing academic performance, with a focus on academic failure and progress. Machine learning, a subfield of artificial intelligence (AI), focuses on designing algorithms that allow computers to identify patterns in data and make predictions or decisions without being explicitly programmed. Closely related, data mining involves uncovering patterns and extracting knowledge from large datasets, frequently employing machine learning techniques to derive meaningful and actionable insights. The analysis involved three main steps: evaluating feature importance, exploring hierarchical feature interactions, and performing classification.

All machine learning and statistical analyses were performed using Python (version 3.9). The primary libraries included scikit-learn (version 1.2.2) for implementing Random Forest, Decision Tree, and Gradient Boosting models, as well as for calculating performance metrics; pandas (version 2.3.1) and numpy (version 1.26.4) for data preprocessing and manipulation; statsmodels (version 0.14.5) for conducting diagnostic tests such as checks for multicollinearity; and matplotlib (version 3.10.0) for generating visualizations and plotting decision trees. Models were trained using an 80:20 train-test split and evaluated over 10 iterations to enhance robustness. Stratified k-fold cross-validation was employed via scikit-learn to address class imbalance. The feature set for both prediction tasks (failure and success) included demographic variables (sex, age, marital status, field of study, year of university entry, gap year, residence status, diploma GPA, total academic GPA in university, employment status, university entry quota) and aggregated trait scores from the questionnaires (personal traits, academic traits, social traits).

We first applied the Random Forest algorithm to assess feature importance, identifying the key variables that most influence academic outcomes. Following this, we constructed decision tree models to understand how the significant features interact hierarchically, highlighting their relative contributions at different levels. Finally, separate classification models were developed to predict academic failure and progress, enabling us to evaluate the predictive capability of the identified features for each outcome.

Analyzing feature importance is crucial for understanding the factors that most influence academic performance, as it helps to identify which features are key drivers of student success or failure. Random Forests is a robust machine-learning algorithm that operates by constructing multiple decision trees. Each tree is trained on a random subset of the data, and the final prediction is made by averaging the results from all trees. This ensemble approach enables Random Forest to capture complex patterns in the data, offering robust predictions [29].

Random Forest is particularly well-suited for feature importance analysis, even with limited sample sizes or imbalanced datasets. This makes it an ideal choice for analyzing the features that affect academic failure and progress, providing reliable insights despite the challenges posed by our dataset [29].

A decision tree functions by dividing the dataset into subsets based on feature values, recursively creating branches that represent decisions or outcomes. Each internal node of the tree signifies a feature, and each branch indicates a decision based on a specific feature threshold. The leaf nodes hold the predicted outcome or class label. Mathematically, the decision tree algorithm employs a process called recursive binary splitting to determine the optimal feature at each node. It assesses the effectiveness of a split using metrics such as Gini impurity or information gain. The aim is to choose features that produce the greatest reduction in uncertainty at each step. Features that result in the most significant decrease in Gini impurity are positioned at the upper nodes of the tree, as they provide the most valuable splits for prediction. This hierarchical structure means that the most influential features are located at the top, helping us understand the key factors that contribute to academic progress or failure.

For the classification task, we selected a range of algorithms to ensure robust and diverse modeling, each offering unique advantages in handling the complexities of predicting academic failure and progress.

Logistic Regression is a simple yet effective linear model, often used for binary classification tasks, providing valuable insights into the relationship between features and the likelihood of academic outcomes. The feature coefficients derived from logistic regression also offer insight into the direction and magnitude of feature influence [30].

K-Nearest Neighbors (KNN) is a non-parametric classifier that predicts the class of a sample based on the majority class of its nearest neighbors. It is effective in capturing local patterns and works well when there are clear clusters in the data. KNN’s simplicity and effectiveness in small, well-defined datasets make it a strong candidate [30].

Naive Bayes classifiers are based on applying Bayes’ theorem with strong independence assumptions. Given that academic performance data often involves a mix of different feature types, such as demographic information, educational history, and behavioral metrics, Naive Bayes’ assumption of feature independence allows it to model the relationships between these variables efficiently [30].

Gradient Boosting is an ensemble method that builds models sequentially, each correcting the errors of its predecessor. It excels at modeling complex relationships and is particularly useful for achieving high accuracy in predictive tasks. It is well-suited to imbalanced datasets, as it can focus on harder-to-classify examples by adjusting the weights of misclassified instances [31].

AdaBoost works by combining weak learners to form a strong classifier. It adjusts the weights of incorrectly classified instances, focusing more on challenging examples. This adaptability makes AdaBoost a practical choice for improving the performance of simpler classifiers, especially when handling outliers or noisy data [32].

LDA is a linear classifier that seeks to find the linear combinations of features that best separate the classes. We chose Linear Discriminant Analysis (LDA) for its ability to efficiently separate classes when the features are normally distributed within each class, a reasonable assumption for many educational datasets [33].

Finally, the MLP, a type of feedforward neural network, was selected for its ability to model complex nonlinear relationships between features. Unlike traditional linear models, MLPs can learn intricate patterns through layers of interconnected neurons, making them suitable for capturing non-linear decision boundaries. Although computationally more intensive, MLPs offer flexibility in modeling and can provide a performance boost with sufficient data [30].

### Ethical considerations

This study was conducted in accordance with the principles outlined in the Declaration of Helsinki. All participants provided written informed consent, and data anonymity was ensured through the use of untraceable identification codes. Additionally, the research protocol was approved by the Ethics Committee of Fasa University of Medical Sciences.

### Feature importance analysis for academic failure and improvement

In this study, we aim to investigate the factors that influence academic improvement and failure, as identified through questionnaires completed by students. One set of questions focuses on the factors contributing to academic failure, while the other examines those that promote academic achievement. By analyzing the responses, we aim to identify key determinants that influence students’ academic trajectories. This analysis will offer valuable insights into how various factors, ranging from individual traits to external influences, impact academic outcomes. It provides a comprehensive understanding of the dynamics behind both academic improvement and failure.

To identify the most important features influencing academic improvement and failure, we utilized the Random Forest algorithm. Although our dataset is relatively small in terms of the number of samples and features, Random Forest still offers several advantages for feature selection. One key reason for choosing this method is its ability to measure feature importance, even in smaller datasets, effectively. Random Forest works by creating multiple decision trees, each focusing on different subsets of the data, and then aggregating their predictions. This allows it to capture complex interactions between variables.

**Fig. 1:**
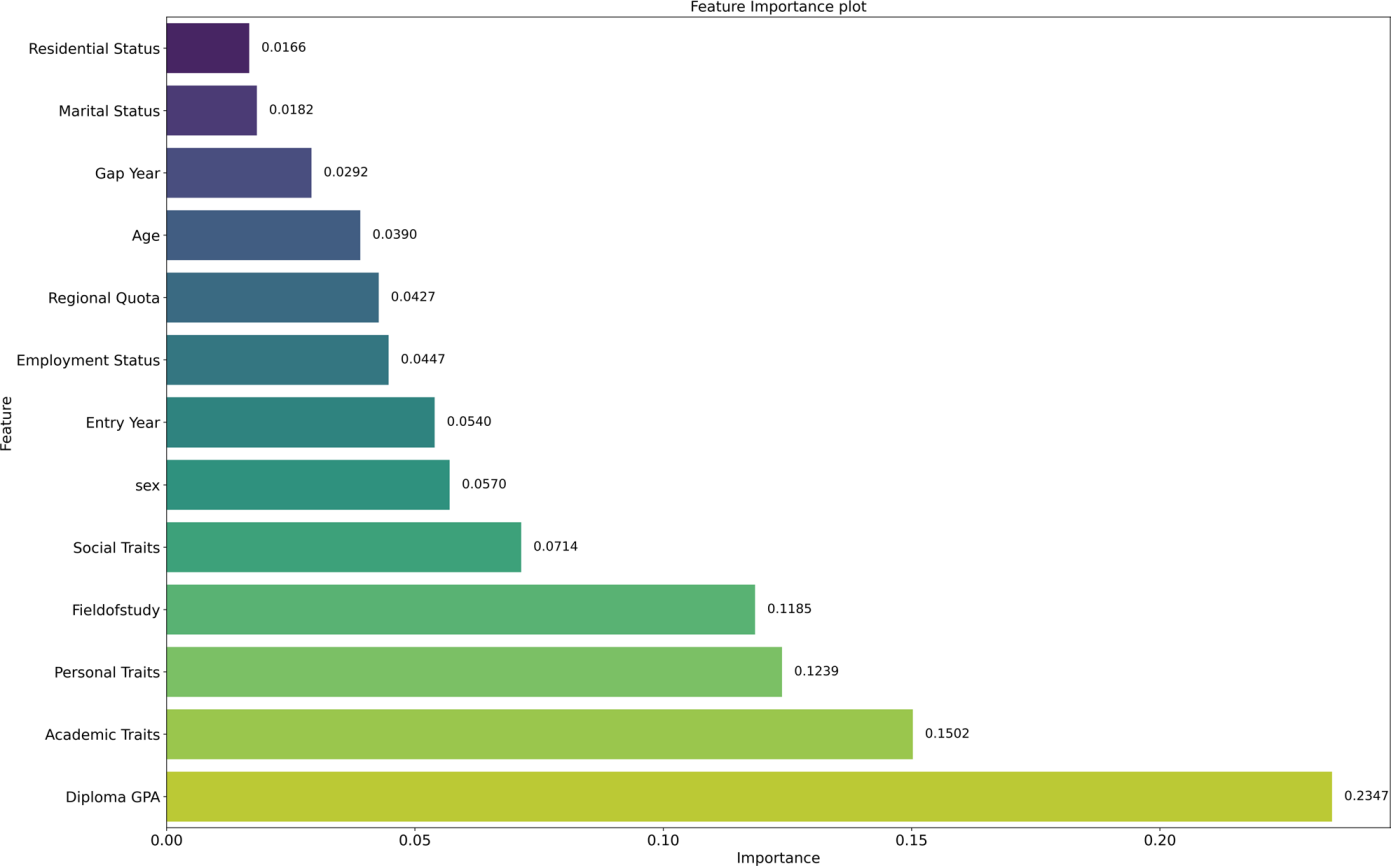
Feature importance analysis of academic failure

The analysis of the feature importance plot for predicting academic performance failure, as shown in Fig. 1, reveals a structure largely governed by academic preparedness and behavioral traits. Diploma GPA stands out as the most influential factor, underscoring the strong link between weaker academic foundations and an increased risk of academic difficulties. Academic traits closely follow, indicating that poor study habits, limited classroom engagement, and a lack of self-discipline significantly increase the likelihood of failure. Likewise, personal characteristics, such as motivation and adaptability, play an essential role, highlighting the protective function of individual resilience in the face of academic challenges.

Additional contributors, such as the field of study, suggest that differences in disciplinary demands and the alignment between students and their academic domains can introduce varying levels of risk. Although social traits are less dominant, they remain meaningful, highlighting how social support, peer interaction, and academic belonging can help buffer against failure. Demographic variables, such as sex, entry year, and employment status, exhibit a moderate influence, suggesting the role of broader life circumstances in shaping vulnerability. Conversely, features such as age, regional quotas, residential status, marital status, and gap year have minimal importance, suggesting their effects on academic failure are primarily indirect and mediated by more central academic and personal characteristics.

**Fig. 2:**
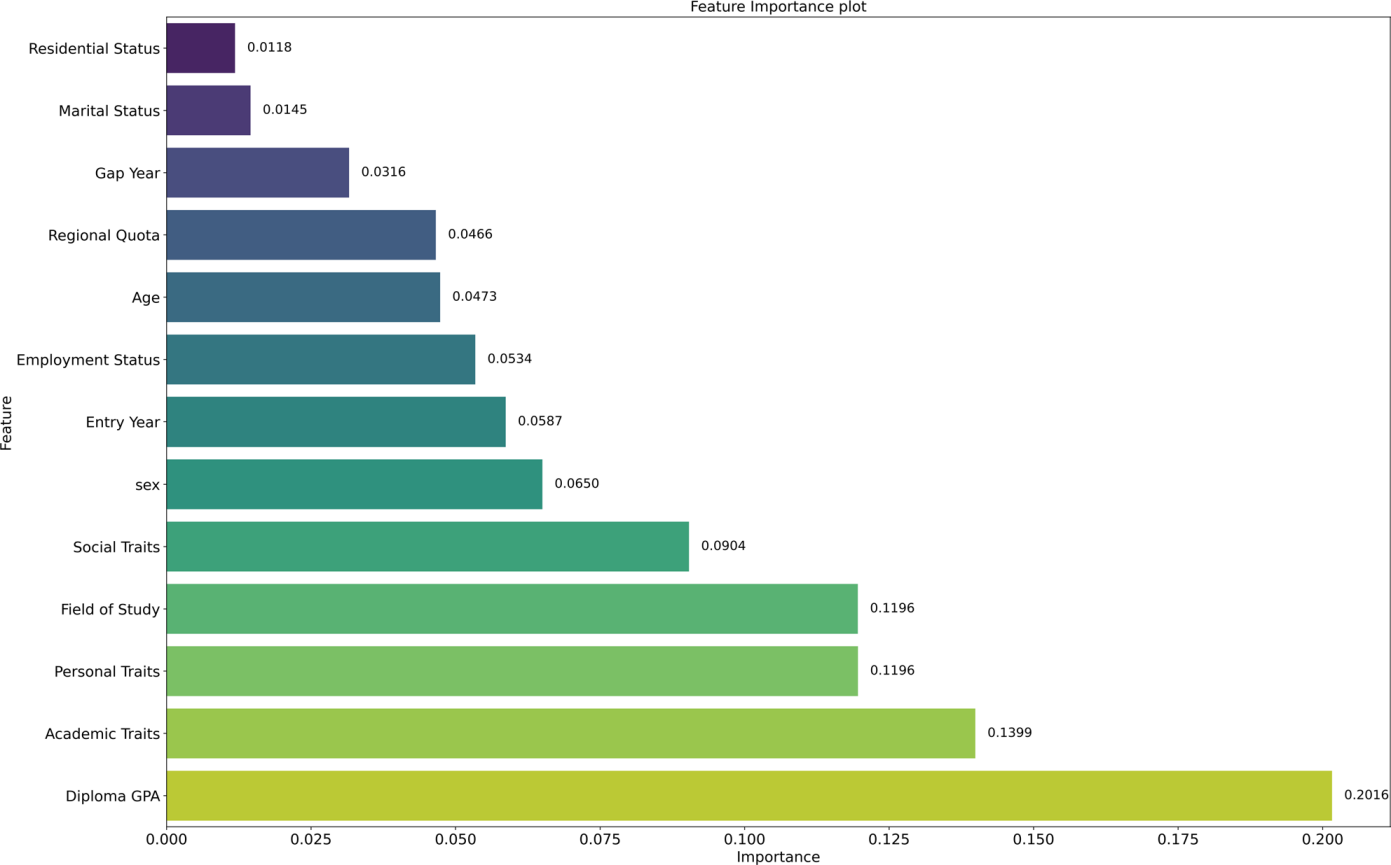
Feature importance analysis of academic success

A remarkably similar pattern emerges in the analysis of academic performance improvement, illustrated in Fig. 2, where academic history and behavioral traits again dominate the model. Diploma GPA once again appears as the most significant factor, this time indicating that prior academic success provides a strong foundation for future achievements. Academic traits, such as consistency, engagement, and time management, also play a critical role, reinforcing the idea that sustainable academic improvement relies on structured effort and disciplined learning habits.

Personal traits and field of study also contribute significantly, reflecting the dual importance of internal motivation and the contextual fit between the student and the academic environment. While slightly less prominent, social traits continue to matter, pointing to the positive influence of social support, collaboration, and extracurricular involvement in promoting growth. Demographic factors, including sex, entry year, and employment status, again exhibit moderate importance, suggesting that timing, life responsibilities, and broader context can shape the likelihood of improvement. At the lowest end of the spectrum, age, regional quota, residential status, marital status, and gap year show limited predictive power, reinforcing the conclusion that while such variables may shape the educational experience, they are not primary drivers of academic advancement.

### Decision tree analysis for academic performance

To further understand the complex factors influencing academic improvement and failure, we employed decision tree analysis. Decision trees are powerful tools for uncovering hierarchical relationships among variables, offering interpretable and visually intuitive insights into the decision-making process. Unlike correlation analysis, which measures the linear relationships between features, decision trees enable us to explore non-linear interactions and identify the most critical factors that split the data into success or failure categories.

This analysis allows us to rank the importance of features while understanding how they interact to predict outcomes. For academic improvement, the decision tree identifies pathways and conditions that lead to positive outcomes, providing actionable insights into fostering academic achievements. Similarly, for academic failure, the decision tree highlights the most significant risk factors and their interactions, offering a framework for targeted interventions to mitigate failure.

By analyzing these trees separately, we can identify unique patterns and distinctions in the pathways that lead to success and failure. These findings not only provide a deeper understanding of the multifaceted nature of academic outcomes but also pave the way for data-driven strategies to enhance educational policies and support systems.

**Fig. 3:**
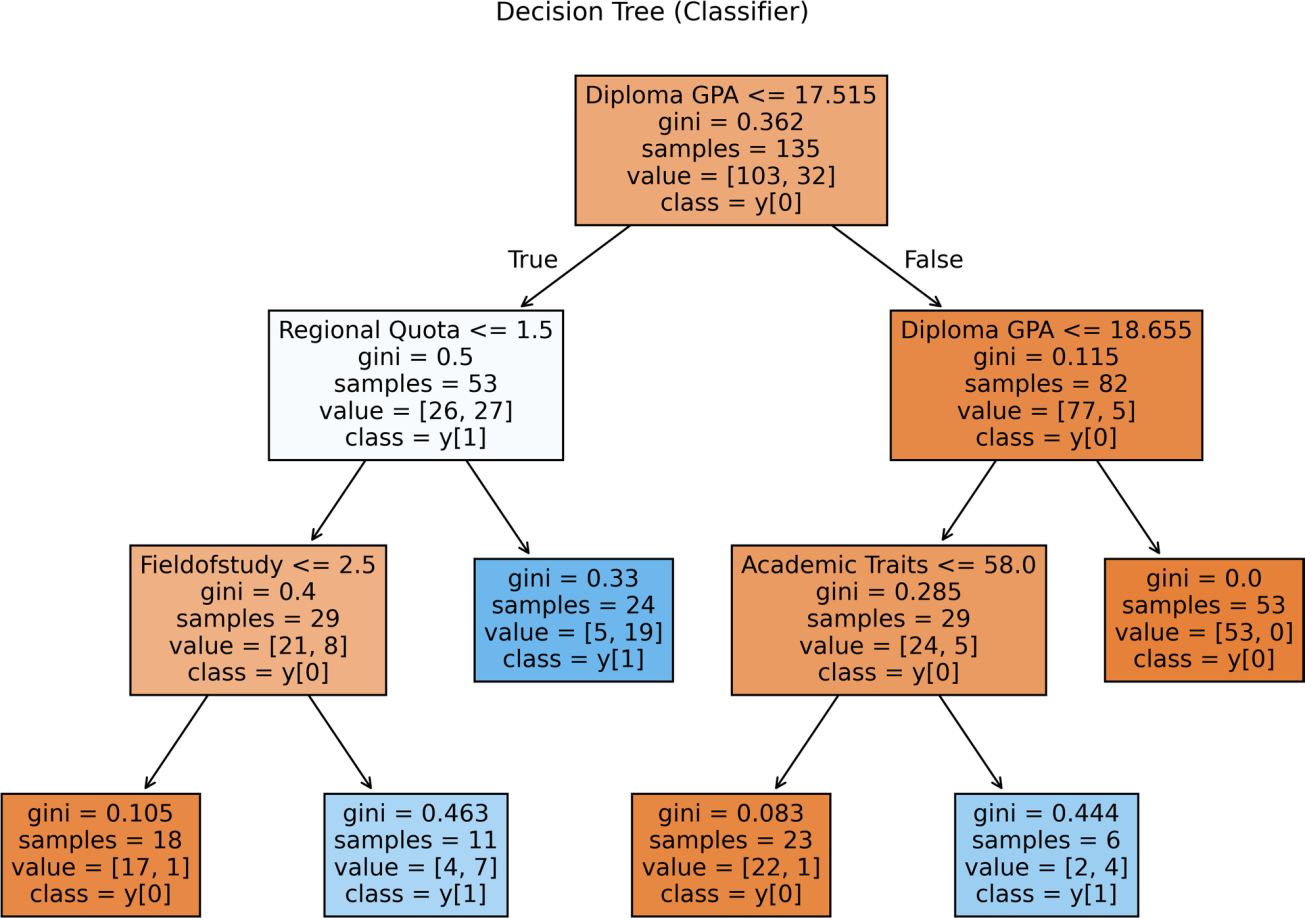
Decision tree analysis for academic failure

The decision tree model elucidates a hierarchical interplay of academic and contextual factors in predicting academic performance failure. In Fig. 3, at the root node, the Diploma GPA (scale: 0–20) serves as the primary discriminator, with a threshold of 17.52 that bifurcates the cohort. Students scoring ≤ 17.52 are further stratified by Regional Quota (1 = high-amenity regions; 2/3 = low-amenity regions). Field of Study subsequently partitions those from high-amenity regions (Regional Quota ≤ 1.50): students in Intelligence (1) or Operating Room (2) programs (≤ 2.50) are classified as stable (no failure), whereas those in Laboratory Science (3) (> 2.50) face elevated failure risk. By contrast, students from low-amenity regions (Regional Quota > 1.50) are directly predicted to experience failure, underscoring socioeconomic disparities as a compounding vulnerability.

For students with a Diploma GPA greater than 17.52, a secondary GPA threshold (18.65) refines predictions. Academic Traits differentiate those in the intermediate range (17.52–18.65). Individuals with traits ≤ 58.00 exhibit resilience (no failure), while those exceeding this threshold paradoxically show a higher risk of failure. The highest-performing cohort (GPA > 18.65) is uniformly predicted to maintain stability, highlighting the protective role of robust prior academic achievement.

**Fig. 4:**
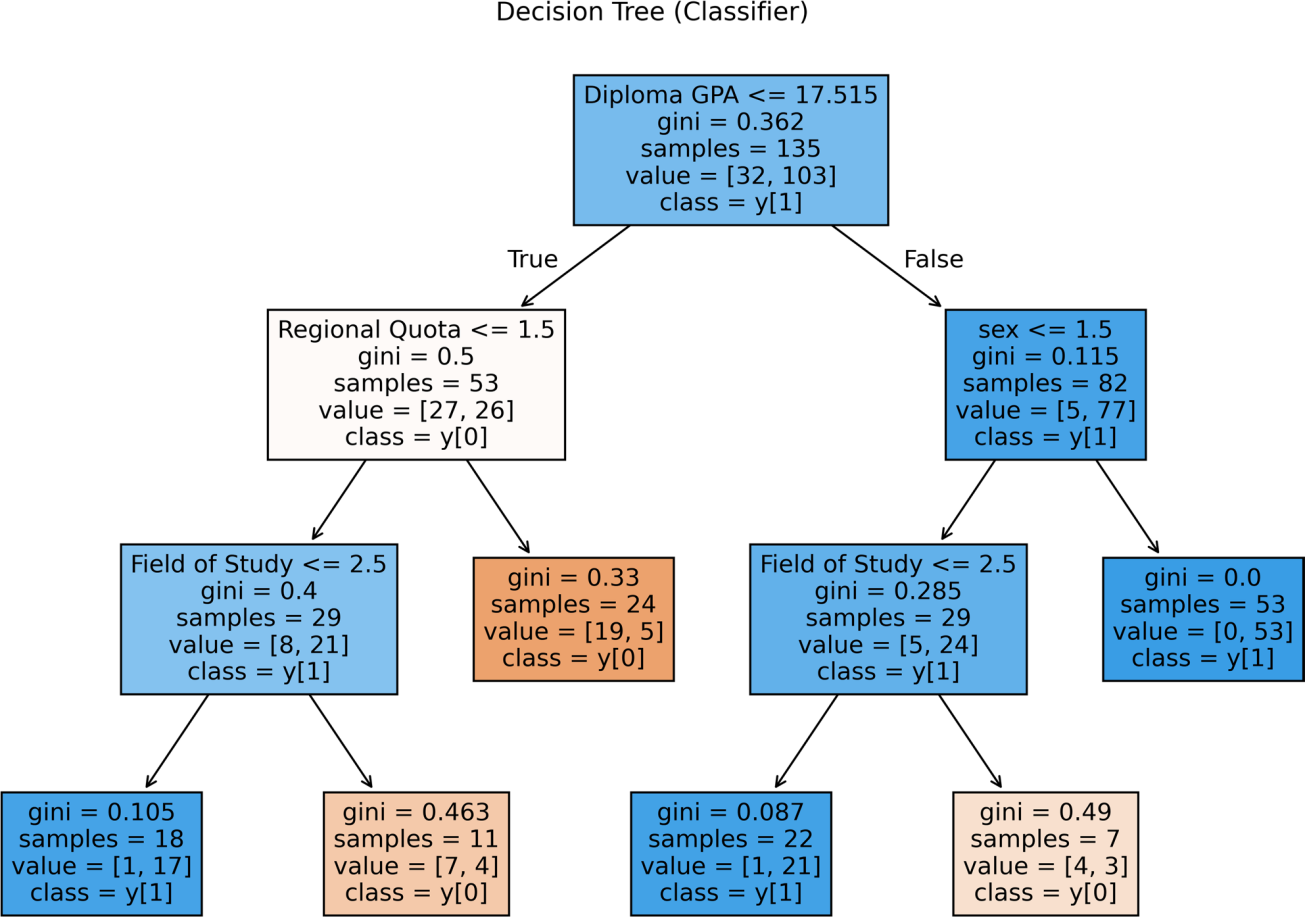
Decision tree analysis for academic success

A discrete decision tree model for academic achievement provides complementary insights. The initial split in Fig. 4 again depends on a GPA ≤ 17.52, with lower performance resulting from bifurcated regional quotas. Students from high-resource areas pursuing careers in operating room/anesthesia sciences tend to achieve success, whereas laboratory science students often stagnate.

Students from lower-resource areas with lower GPAs are predicted to experience no direct educational progress. For a GPA above 17.52, gender dominates the results: female students generally succeed regardless of their major, while male students tend to progress only in the operating room and anesthesia disciplines. This structure highlights gender-based resilience, with female students benefiting from systemic or adaptive advantages across disciplines, while male success depends on domain-specific environments.

### Analysis of predictive models for academic performance

This cross-sectional study utilizes machine learning models to predict academic failure or improvement in students, while identifying critical factors that influence these outcomes. By evaluating models through metrics such as Accuracy, Precision, Recall, F1-Score, AUC, and G-Mean, the analysis reveals how these measures reflect student-specific risks, progress, and the reliability of predictors shaping their academic journeys.

Accuracy quantifies the overall correctness of predictions by measuring the proportion of correctly classified students, both those experiencing academic failure and those improving, relative to the total population. While it provides a broad performance snapshot, it can be misleading in imbalanced datasets. For instance, if the majority of students are improving, a high accuracy score may mask the model’s failure to identify those at risk of failure. As a result, accuracy serves as a useful preliminary benchmark but lacks reliability in detecting vulnerable students [34].

Precision evaluates the model’s ability to minimize false positives, indicating the proportion of students predicted as “at risk” or “improving” who truly belong to those categories. While high precision ensures fewer misclassifications, overly precise models may exclude borderline cases, students with fluctuating performance who still require intervention [35].

Recall measures how effectively the model identifies all students experiencing academic failure or improvement, reducing the likelihood of overlooked cases. A high recall is critical for ensuring that no at-risk student is left unidentified, making it a key metric for early intervention strategies [35].

The F1 score balances precision and recall, providing a single metric that ensures the model does not over-prioritize one at the expense of the other. It is beneficial when both false positives and false negatives carry significant consequences, preventing extreme conservatism or over-optimism in classification [34].

AUC (Area Under the ROC Curve) assesses the model’s ability to distinguish between declining and improving students across all classification thresholds. A higher AUC indicates better separation, meaning the model effectively ranks students by risk severity. This is especially valuable for prioritizing students with compounding risk factors, such as low diploma GPA and socioeconomic disadvantages. Unlike accuracy, AUC remains robust in the presence of class imbalances, making it a reliable performance measure for academic datasets [34].

G-Mean (Geometric Mean) evaluates the model’s balanced performance across both classes by calculating the geometric mean of sensitivity (recall) and specificity (true negative rate). A high G-Mean ensures that the model does not disproportionately favor majority groups (e.g., high-performing students) while neglecting minority groups (e.g., at-risk students from underprivileged backgrounds). This metric is crucial for ensuring equitable intervention strategies, enabling fair support for both struggling and excelling students without bias [36].

To ensure robust generalizability, the dataset was partitioned into an 80% training set and a 20% test set, with performance metrics averaged over 10 independent repetitions of this split. This stratified approach mitigates sampling bias and provides stable estimates of model efficacy, particularly critical given class imbalances in both failure (minority class) and improvement (majority class) prediction tasks.

Table 1 presents the classification results for academic failure, highlighting Random Forest's top performance, while Table 2 details the predictions for academic success, with Gradient Boosting leading in both accuracy and F1-score. In both tables, bold values denote the highest performance achieved for each evaluation metric among the evaluated classifiers. The classifiers demonstrate varied effectiveness in identifying students at risk of academic failure. Among them, Random Forest stands out with the highest accuracy (90.74%), F1-score (76.19%), and AUC (96.19%), showcasing its robust generalization capabilities across imbalanced classes. Its balanced precision (80%) and recall (72.73%) indicate a strong ability to minimize false positives while capturing true failure cases.

**Table 1:** Classification result for academic failure

Classifier	Accuracy	Precision	Recall	F1-Score	AUC	G-Mean
Logistic Regression	88.88	72.72	72.72	72.72	94.50	8.22
Decision Tree	74.07	38.46	45.45	41.66	63.42	6.082
Random Forest	**90.74**	**80**	**72.72**	**96.19**	**96.19**	**8.32**
K-Nearest Neighbors (KNN)	74.07	28.57	18.18	22.22	66.06	4.00
Naive Bayes	85.18	71.42	45.45	55.55	91.96	6.58
Gradient Boosting	87.03	66.66	72.72	69.56	94.71	8.12
AdaBoost	87.03	66.66	72.72	69.56	93.86	8.12
Linear Discriminant Analysis (LDA)	88.88	66.66	90.90	76.92	94.92	8.96
Multilayer Perceptron (MLP)	88.88	69.23	81.81	75	90.69	8.61

**Table 2:** Classification result for academic success

Classifier	Accuracy	Precision	Recall	F1-Score	AUC	G-Mean
Logistic Regression	83.33	90.47	88.37	89.41	89.64	7.49
Decision Tree	83.33	86.95	93.02	89.88	69.23	6.50
Random Forest	88.88	89.36	97.67	93.33	92.38	7.29
K-Nearest Neighbors (KNN)	70.37	78.72	86.04	82.22	60.78	2.79
Naive Bayes	81.48	83.67	95.34	89.13	86.25	5.09
Gradient Boosting	**90.74**	**93.18**	**95.34**	**94.25**	**93.44**	**8.32**
AdaBoost	88.88	89.36	97.67	93.33	82.45	7.299
Linear Discriminant Analysis (LDA)	85.18	97.29	83.72	90	93.65	8.72
Multilayer Perceptron (MLP)	75.92	89.47	79.06	83.95	89.21	7.093

Linear Discriminant Analysis (LDA) distinguishes itself with an exceptional recall rate (90.91%) and G-mean (8.96), reflecting its high sensitivity to true failure instances. However, its moderate precision (66.67%) indicates a relatively high false positive rate. Gradient Boosting and AdaBoost perform comparably in this task (F1 ~69.57%, AUC ~94), though AdaBoost slightly trails in AUC (93.87 vs. 94.71), likely due to its sensitivity to noisy data from iterative weighting.

Shifting focus to academic improvement prediction, Gradient Boosting clearly dominates, achieving the highest accuracy (90.74%), F1-score (94.25%), and AUC (93.45). This underscores its strength in modeling the complex, non-linear patterns and sequential dynamics of academic progression. AdaBoost and Random Forest follow closely behind (F1 = 93.33%), with AdaBoost attaining a notably higher recall (97.67%) but a lower AUC (82.45%), suggesting an overemphasis on difficult instances at the expense of ranking stability. LDA again performs strongly in precision (97.30%) and G-mean (8.72), making it well-suited for scenarios that prioritize minimizing false positives, such as targeted support interventions. Logistic Regression and Naive Bayes yield moderate results (F1 = 89.41–89.13), with Naive Bayes achieving a high recall (95.35%) but sacrificing precision (83.67%). The Decision Tree, while achieving a competitive F1-score (89.89%) in the improvement task, surpasses its performance in failure prediction, likely due to the simpler structure of decision boundaries in this context. However, its relatively low AUC (69.24) and G-mean (6.50) indicate weaknesses in effectively ranking true positive cases. 

**Fig. 5:**
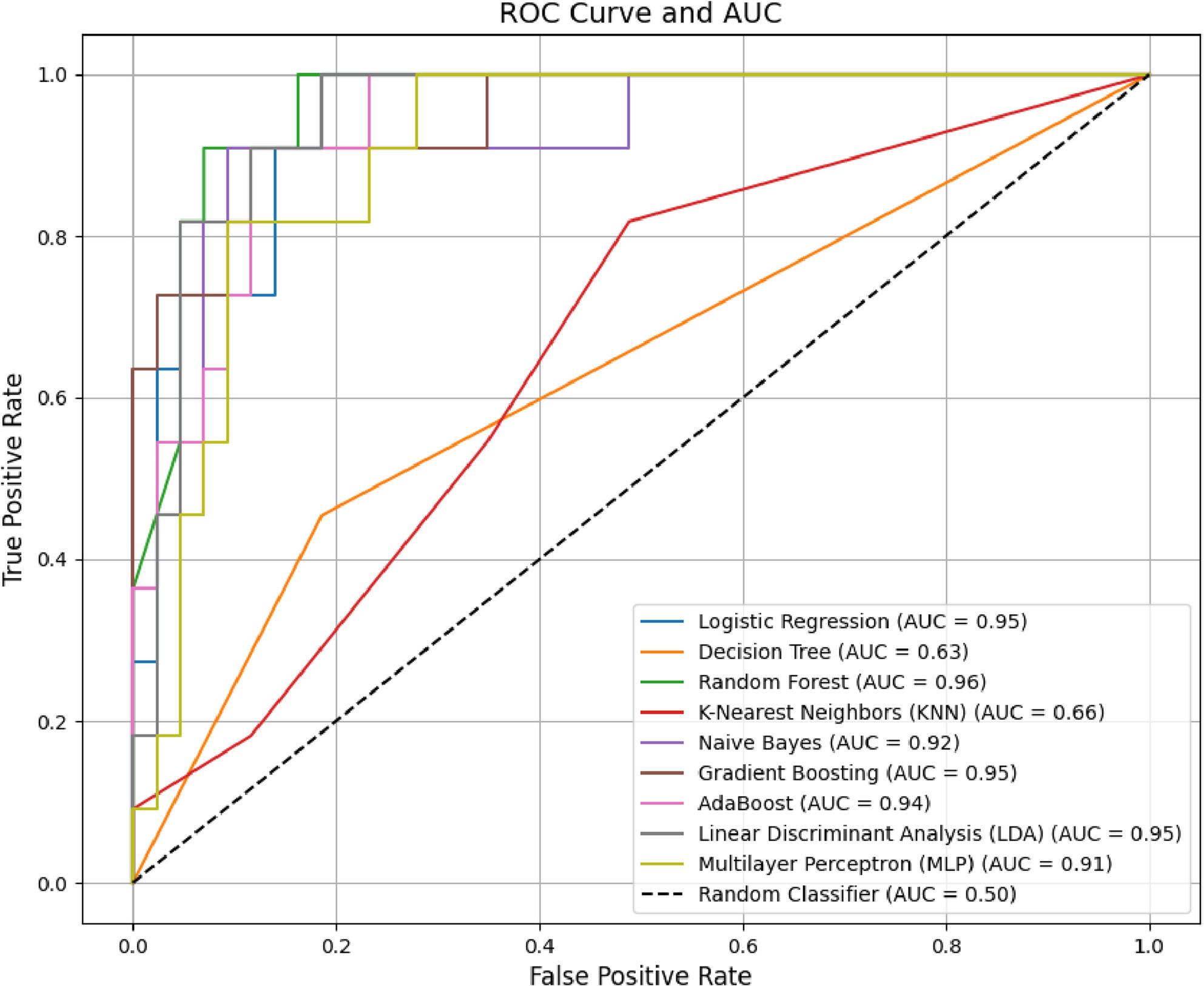
ROC curves for classifiers predicting academic failure with AUC > 50

**Fig. 6:**
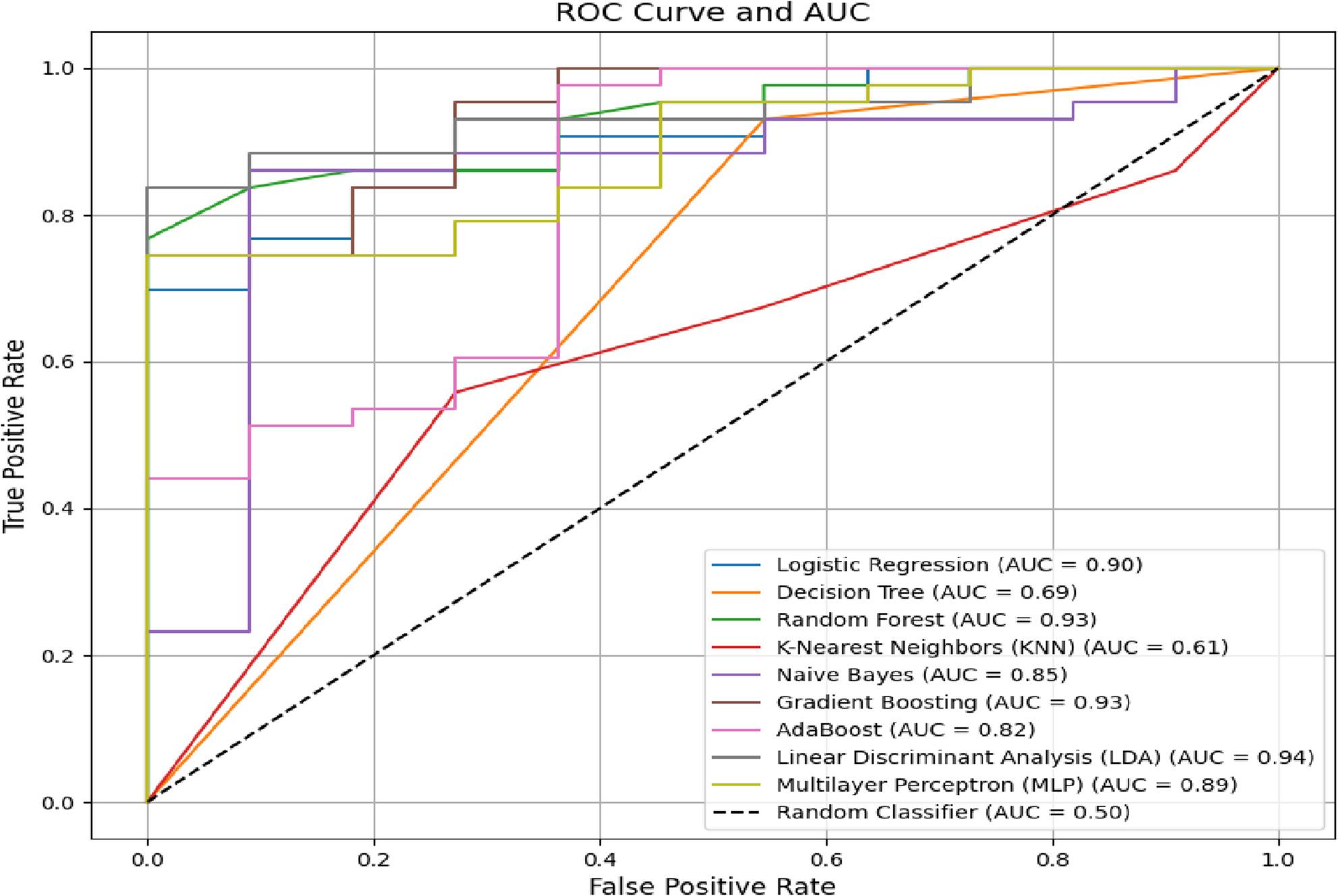
ROC curves for classifiers predicting academic improvement with AUC > 50

Below, in Figures 5 and 6, we present the Receiver Operating Characteristic (ROC) curves for classifiers with an AUC greater than 50. These curves illustrate the trade-off between true positive rates (sensitivity) and false positive rates (1-specificity) at varying classification thresholds.

For failure prediction (Fig. 5), the Random Forest (AUC = 96.19) and Linear Discriminant Analysis (AUC = 94.93) exhibit near-perfect separability, with steep early increases in true positive rates, underscoring their utility for prioritizing at-risk students. In contrast, the Decision Tree (AUC = 63.42) and K-Nearest Neighbors (AUC = 66.07) show flatter trajectories, reflecting limited discriminative power.

For improvement prediction, Fig. 6 shows that Gradient Boosting (AUC = 93.45) and AdaBoost (AUC = 93.33) dominate the upper-left quadrant, achieving high true positive rates at minimal false positive costs, a critical feature for institutions targeting efficient resource allocation. The stark divergence in curved shapes between tasks highlights the distinct mechanistic pathways governing failure (contextually driven) versus improvement (behaviorally mediated). These visualizations corroborate the quantitative metrics, reinforcing the superiority of ensemble methods in modeling academic trajectories and the need for task-specific algorithm selection.

## Discussion

This study contributes to the application of machine learning in educational research by providing data-driven insights to guide targeted interventions and policy refinements in paramedical education. By employing ensemble models, we observed that machine learning models can achieve strong predictive performance for academic success and failure within this single-center cohort. Notably, Random Forest showed the strongest performance for identifying at-risk students, whereas Gradient Boosting performed best for forecasting academic success. These outcomes align with previous work underscoring the robustness of ensemble methods in educational settings [37, 38]. However, direct comparisons of performance metrics across studies should be interpreted cautiously, as published results may differ substantially due to variations in sample size, outcome definitions, feature sets, class balance, and validation strategies. A central finding of this study is the dominant role of high school GPA in predicting both academic failure and success, aligning with national [39] and international evidence [40–42] that pre-university achievement exerts a strong influence on higher education trajectories. This underscores the importance of heavily weighing high school records in admissions decisions and identifying at-risk students early for personalized support. In the Iranian context, however, this predictor’s influence is amplified by distinctive systemic factors, including the highly competitive national entrance examinations and quota systems (regional and martyr-based), which are less common in Western educational models. These contextual differences suggest that predictive models and intervention strategies developed in international settings may require adaptation to the Iranian educational system. From a policy and practice perspective, this highlights the importance of context-sensitive admission policies and early, targeted academic support for students identified as at risk within the Iranian medical education framework. Beyond predictive accuracy, recent studies suggest that AI-driven educational tools can also support students’ motivation, emotional engagement, and perceived support, highlighting the importance of integrating predictive models with affective and motivational interventions [43, 44].

Iranian research frequently emphasizes variables such as parental education, gender dynamics, socioeconomic status, dormitory living, employment, and marital status as key determinants of academic performance. Entrance quotas and diploma grades often exert a stronger moderating effect on failure risk in Iran than reported in global studies. In contrast, international investigations in paramedical and medical fields tend to highlight constructs such as self-efficacy, self-regulated learning, motivation, institutional support, social resources, and socio-cultural adaptation. Our findings also suggest preliminary hierarchical interactions—such as those involving quota systems—that may contribute to regional disparities or gender-related differences in outcomes, particularly within Iran’s collectivist and resource-constrained educational settings, especially in southern regions where dormitory-based living is common [45, 46]. Nonetheless, these observations—including apparent gender-related patterns (e.g., divergent pathways)—should be regarded as exploratory, given the high instability of decision trees on small datasets and the risk of overfitting. The decision-tree components of our ensemble models enhance interpretability by visually mapping potential interactions and contextual factors (e.g., quotas or study fields). However, these findings require cautious interpretation and further validation through larger samples to ensure reliability. Greater clarity in this area could inform more targeted interventions, from mentorship initiatives to equitable admission reforms. Moreover, our focus on educational and institutional factors reinforces the importance of improving teaching approaches and strengthening relationships between professors and students to enhance academic performance and retention.

### Limitations

This study has several limitations that should be considered when interpreting the findings. First, the small, non-random sample of 135 students from a single institution (Fasa University of Medical Sciences, Iran) limits the generalizability of the results and may introduce selection bias. Second, the cross-sectional design restricts causal inference, and the use of self-reported questionnaires may be affected by social desirability and recall bias. Third, the limited dataset size raises concerns regarding overfitting and model instability, particularly for decision tree–based methods, which are sensitive to small-sample variations and may yield speculative or unreliable split patterns (e.g., by gender or quota). Accordingly, the near-perfect discrimination suggested by some AUC values should be interpreted cautiously and requires confirmation through external validation in independent cohorts. Future research should therefore involve larger, multi-site, longitudinal cohorts; incorporate fairness audits (e.g., by gender or quota type); and test the models’ applicability across disciplines to strengthen the evidence base for AI-driven, equitable, and data-informed educational strategies. Given that AI-based prediction systems may benefit many students while unintentionally disadvantaging some groups, fairness-aware evaluation is essential prior to real-world implementation [27].

## Conclusion

This study aimed to employ machine learning models to predict academic performance and identify factors associated with success and failure among paramedical students. The findings suggest that, among demographic variables, high school GPA is a strong predictor of academic outcomes. In addition, questionnaire-derived educational factors appeared to be the most influential domain compared with social and personal factors. These results may help inform educational planning and early identification of students who could benefit from targeted support. Among the evaluated models, Random Forest showed the best performance for identifying students at risk of academic failure, while Gradient Boosting performed best for predicting academic success. However, given the single-center, cross-sectional design and the use of convenience sampling, the findings should be interpreted cautiously and may not be generalizable to other student populations. Future studies should validate these models in larger, multi-center cohorts using probability-based or consecutive sampling to confirm robustness and generalizability.

## Data Availability

The datasets used and/or analyzed during the current study are available from the corresponding author on reasonable request.

## Abbreviations

RF: Random Forest
DT: Decision Tree
KNN: K-Nearest Neighbors
MLP: Multi-Layer Perceptron
LDA: Linear Discriminant Analysis
SVM: Support Vector Machine
GPA: Grade Point Average
